# Supermarket promotions in Western Sweden are incompatible with Nordic dietary recommendations and differ by area-level socioeconomic index

**DOI:** 10.1186/s12889-023-15729-1

**Published:** 2023-04-28

**Authors:** Melissa Mjöberg, Lauren Lissner, Monica Hunsberger

**Affiliations:** School of Public Health and Community Medicine, Institute of Medicine, Sahlgrenska Academy, University of Gothenburg, Gothenburg, Sweden

**Keywords:** Food environment, Food advertising, Nutrition guidelines, Sweden, Supermarket, Healthy diet, Socioeconomic area

## Abstract

**Background:**

Large supermarket chains produce weekly advertisements to promote foods and influence consumer purchases. The broad consumer reach of these ads presents an opportunity to promote foods that align with dietary recommendations. Thus, the aim of this study was to investigate the health quality of supermarkets’ weekly food promotions in a large region of Sweden with attention to more and less advantaged socioeconomic index areas.

**Methods:**

Analysis of weekly advertisements from 122 individual stores, representing seven chains, was carried out in a large region of Sweden from 2–29 March in 2020. Food promotions were divided into categories according to the Nordic Nutrition Recommendations and World Health Organization Regional Office for Europe’s nutrient profile model, and defined as ‘most healthy’, ‘healthy’, ‘unhealthy’ and ‘most unhealthy’. A mean socioeconomic index was used to classify each store location to determine whether proportions of the ‘most unhealthy’ foods differed between more advantaged and more disadvantaged socioeconomic index areas.

**Results:**

In total, 29,958 food items were analyzed. Two-thirds of promotions belonged to the food groups considered ‘most unhealthy’ and ‘unhealthy’. In the ‘most unhealthy’ food group ‘sugar-rich beverages and foods’ constituted approximately 23.0% of the promotions. Food promotions had 25% increased odds to be from the ‘most unhealthy’ group (odds ratio 1.25, confidence interval 1.17, 1.33) in more disadvantaged socioeconomic index areas. This association could be explained by the supermarket chain the stores belonged to.

**Conclusions:**

Our findings indicate that Swedish supermarkets promote a large proportion of unhealthy foods as classified by the Nordic Nutrition Recommendations. We also observe that certain national supermarket chains tend to locate their stores in more disadvantaged areas and promote a greater proportion of unhealthy foods in their weekly advertisements compared to the more advantaged areas. There is an urgent need for supermarkets to shift promotions toward healthier food items.

**Supplementary Information:**

The online version contains supplementary material available at 10.1186/s12889-023-15729-1.

## Background

The supermarket is a primary venue for grocery shopping, where various external factors influence consumer buying behavior, e.g. price, promotion, nutritional information, quality, freshness, use of health claims, placement and labelling [1–5]. Consumers make multiple food choices at each visit and when exposed to much information concurrently, choices tend to become less conscious and increasingly vulnerable to marketing influences [1, 6].

In Sweden, both the total energy consumption and the consumption of refined products have increased per capita since 1980 [7]. Above all, the young population consumes too little fruit, vegetables and fiber, and excessive amounts of energy-dense foods and beverages high in fat, salt and sugar [8]. The food environment is dominated by formal markets that offer Swedish consumers a variety of food all year round at low prices. The availability of both healthy and unhealthy foods is high, but marketing of unhealthy foods has been reported to be particularly frequent [9]. For less affluent households, it is problematic because they spend a larger proportion of the household budget on food [10] and are often extra price sensitive [11, 12]. Extensive marketing of unhealthy foods could increase already existing health gaps since they often cost less per calorie than healthy foods [13, 14].

Supermarkets in Sweden, and many other countries across all continents [15, 16], use weekly advertisement sheets (ad-sheets) to promote what is on weekly sale. These ad-sheets have a wide reach [17] and are often available in both physical and digital form. In addition, they probably reflect in-store sales [18]. As the general Swedish population has high digital competence, well above the European Union average [19], it is important that both the physical and digital consumer food environment is healthy. It may be even more important to study the healthiness of food marketing at a time when Sweden is experiencing high food inflation [20] as they are usually marketed at a discount.

The Nordic Nutrition Recommendations (NNR) are produced periodically by the Nordic Council of Ministers [21] and communicated in the Swedish context by the Swedish Food Agency [22]. The latest NNR from 2012 propose an increased intake of vegetables and pulses, fruit and berries as well as dietary fiber and limited consumption of red meat, discretionary and refined foods [21]. From a public health perspective, food campaigns by the Swedish retail should be compatible with these recommendations.

To the authors’ knowledge, no study in Sweden has evaluated the health quality of supermarkets’ weekly ad-sheet promotions. However, studies documenting unhealthy supermarket food promotions have been conducted in United States [17, 23–25], Australia [18], the Netherlands [26, 27], and Brazil [16] together with one international comparison of twelve countries [15]. Thus, the aim of this study was to investigate the health quality of supermarkets’ weekly food promotions in a large region of Sweden with attention to more and less advantaged socioeconomic index areas.

## Methods

### Study design

This cross-sectional study is based upon data collected from 2–29 March in year 2020 from weekly online ad-sheets published by seven supermarket chains across a large region in Western Sweden.

### Data collection

The supermarket chains were selected based on leading market share in 2019. All individual supermarkets representing the chains were identified using Google Maps. From these leading chains, all online versions from 122 individual supermarkets across the region were compared to the printed advertisements and found to be identical. The seven chains (coded A-G), number of individual stores per chain and their promotions are presented in Table 1.

**Table 1:** Number of promoted items in each chain and individual store

Chain	Market share of the supermarket conglomerates (%)	Individual stores per week (*n* = 122)	Promoted food items over 4 weeks (*n* = 29,958)
A	51.5^1^	11	2252
B	17.8^a2^	17	3264
C		39	7592
D	16.9^1^	7	1302
E	7^1^	4	800
F	4.7^1^	28	7644
G	2.1^1^	16	7104

^a^Chains B and C belong to the same conglomerate and the available data and their market share in 2019 is combined [28, 29]

^1^Data from 2019 [29]

^2^Data from 2017 [30, 31]

### Content analysis

In order to analyze the content of the ad-sheets, food categories from NNR [21] were used to correctly classify the ads as ‘most healthy’, ‘healthy’, ‘unhealthy’ and ‘most unhealthy’. Table 2 illustrates the content analysis template that is based on NNR version 2012 [21], with slight modifications of some food categories for the purpose of this study. The Swedish Food Agency uses an arrow, which we have reproduced here, to indicated that consumers should aim to exchange items from the ‘unhealthy’ to the ‘healthy’ group. E.g. from refined to wholegrain cereals and from butter-based to vegetable oil-based fat spreads [22].

**Table 2:**
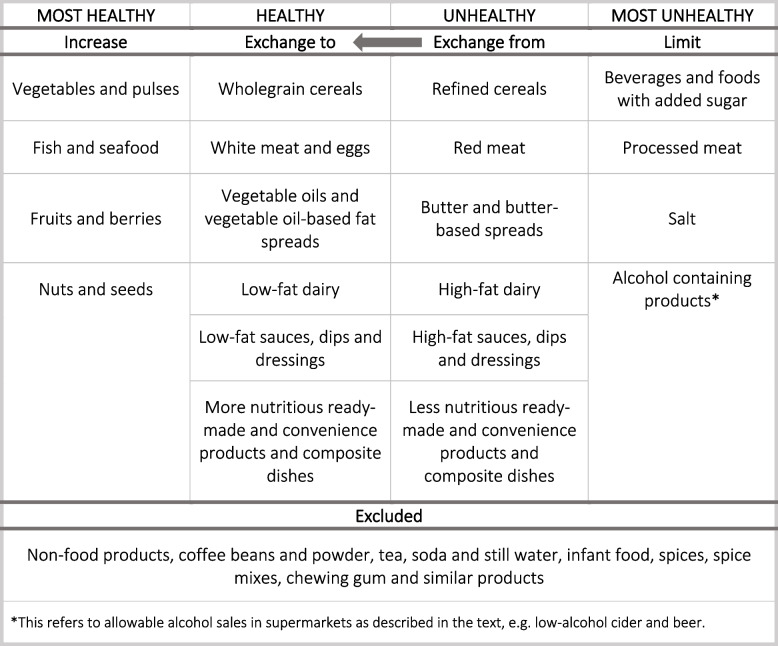
Content analysis template with arrow referring to a recommended replacement of ‘unhealthy’ to ‘healthy’ foods [21]

^*^This refers to allowable alcohol sales in supermarkets as described in the text, e.g. low-alcohol cider and beer

The national authority’s front of package “keyhole” symbol criteria for foods of high nutritional quality as well as the Swedish Food Agency's Code of Statutes [32] were used as steering documents to be consistent with ambiguous food items. The food classification was performed by a food scientist (MM). Food categories and inclusion conditions used during the data input process are described in Additional file 1. To differentiate between unprocessed and processed meat, definitions from the Swedish Food Agency were used [33]. World Health Organization Regional Office for Europe’s (WHO Europe) nutrient profile model [34] was used to add food categories that were missing in NNR’s model in order to categorize all food promotions. Beverages with any alcohol content are included in the food group ‘most unhealthy’ in this study. According to Swedish law, alcohol products containing 3,5% alcohol or less can be sold in supermarkets, all other alcohol must be purchased at the state monopoly and are therefore not promoted in the ad-sheets [35, 36].

### Data input and variables

Food items promoted more than one time in the same ad-sheet were recorded individually to represent the number of visual promotions. Non-categorizable promotions were allocated to an “excluded” category. Non-food items were not included in this analysis.

### Area-level socioeconomic classification of stores

Socioeconomic characteristics of stores were classified applying the socioeconomic index (SEI) from Statistics Sweden to different geographical areas within the region of Western Sweden [37]. The SEI ranges from 0–100 percent and reflects socioeconomic disadvantage based on three indicators: proportion of inhabitants with 1) low economical standard, 2) basic education, and 3) financial support and/or unemployment. The index calculates the mean of the three proportions to get a percentage for each area, with a higher SEI value indicating more disadvantaged socioeconomic conditions [38]. The mean SEI was used as a threshold to compare more versus less affluent SEI areas.

### Method of analyses

The proportion of promoted food categories by health quality (four groups ranging from ‘most healthy’ to ‘most unhealthy’) were described in terms of frequency, percent, and 99% confidence interval (CI). Comparison between promotion of ‘most unhealthy’ foods in more advantaged versus more disadvantaged neighborhoods were also described.

Pearson’s Chi-square was used to test for differences between more and less advantaged areas and promotion of food belonging to the four health groups. Binary logistic regression was used to test if there were higher odds of most unhealthy food to be promoted in more disadvantaged neighborhoods. A multivariable logistic regression analysis was used to test if chain was a confounding factor in the association between SEI area of the store and promotion of most unhealthy food. *P* < 0.01 was considered as significant. IBM SPSS Statistics 27 was used for all data analyses.

A sensitivity analysis was performed to test the robustness of the findings by removing the one chain with no stores in more disadvantaged areas.

### Ethics

The study did not require ethical permission because human subjects were not involved. The chains are not identified in this article.

## Results

### Promoted foods by healthfulness and food categories

**Fig. 1:**
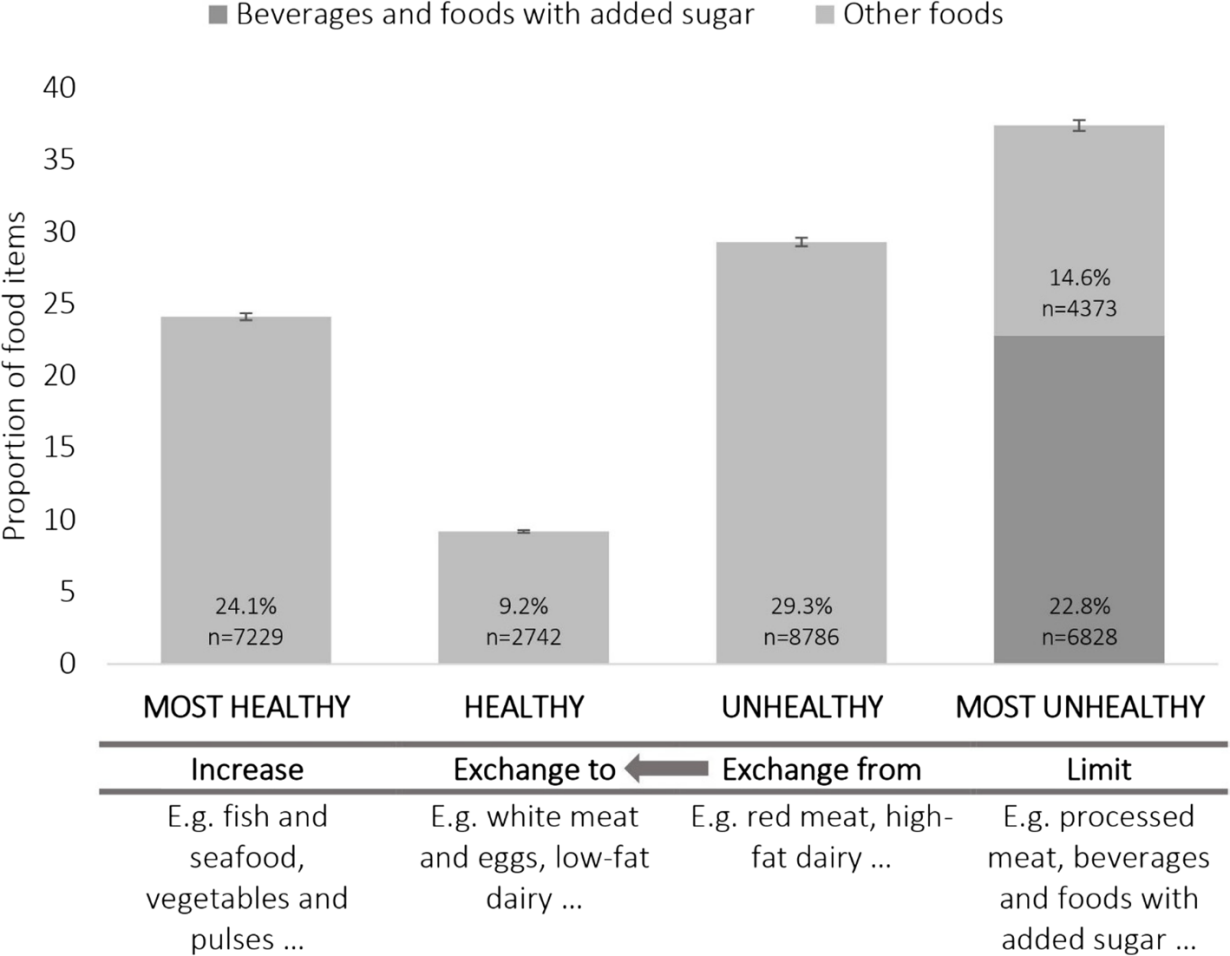
Proportion of promoted foods across the four health groups with corresponding recommendations

After four weeks, 488 ad-sheets containing 42,139 food items were analyzed. After excluding the promotions that could not be categorized by health quality, 71.1% (*n* = 29,958) of the individual food promotions remained for analysis. Of the total promoted foods, 37.4% were categorized as ‘most unhealthy’. See Fig. 1 for proportions of promoted foods across the four health groups. To view all food categories included in each food group we refer to table 2.

Of the foods promoted, 66.7% belonged to the two unhealthy food groups. The most promoted items in these groups were ‘beverages and foods with added sugar’ (22.8%), ‘processed meat’ (11.7%), ‘high fat dairy’ (9.5%) and ‘red meat’ (7.9%). The least promoted unhealthy item was ‘alcohol containing products’ (0.4%). In the healthy and most healthy food groups, the most commonly promoted items were ‘vegetable and pulses’ (10.4%), ‘fruit and berries’ (6.8%) and ‘fish and seafood’ (6.6%). The least promoted items in these groups were ‘vegetable oil and oil-based fat spreads’ (1.2%), ‘low fat dairy’ (0.7%), ‘healthier ready-made meals’ (0.7%), ‘nuts and seeds’ (0.3%) and ‘healthier sauces, dips and dressings’ (0.1%).

### Proportion of promoted unhealthy food in stores located in more disadvantaged and more advantaged areas

**Fig. 2:**
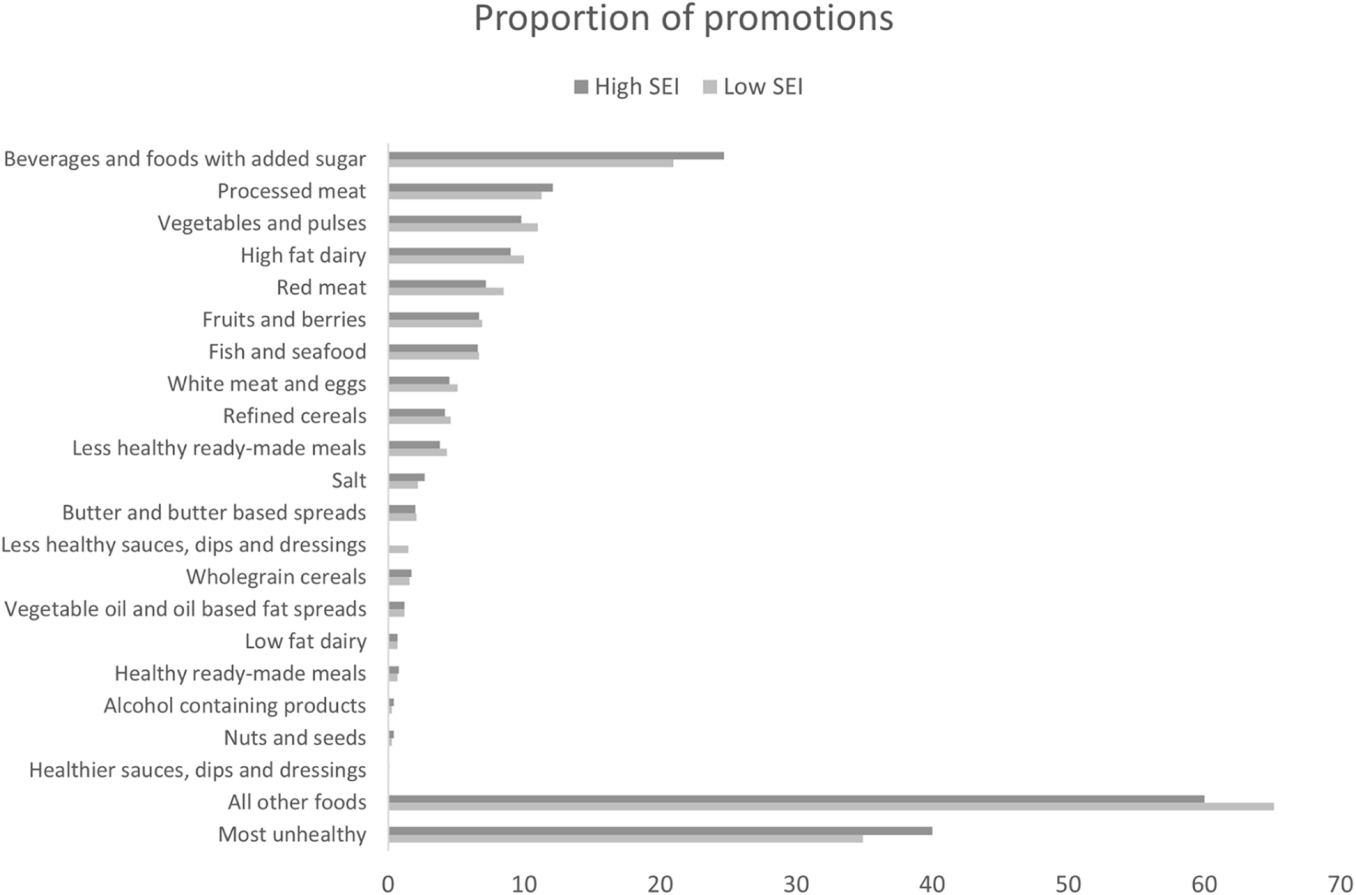
Promoted food categories shown by more disadvantaged and more advantaged store SEI area

In this study, the SEI ranged from 2.9% (most advantaged SEI area) to 42.2% (most disadvantaged SEI area). Considering the 122 store areas, the mean SEI and SD were 11.2 (± 6.3). There was a difference in mean proportion of advertised food categories between more advantaged and more disadvantaged neighborhoods, where the unhealthiest food categories were promoted to a higher extent in the more disadvantaged neighborhoods. The mean proportion of promoted most unhealthy food in stores located in more disadvantaged areas was 40.0%, and in more advantaged areas the mean proportion was 34.9%. The food category that was promoted most frequently was ‘beverages and foods with added sugar’. In the more advantaged SEI areas, the food category had a proportion of 14.7% and in the more disadvantaged SEI areas, the proportion was 17.7%, see Fig. 2.

Food promotions in more disadvantaged neighborhoods had 25% increased odds to be in the ‘most unhealthy’ group (*p* < 0.001). However, adjusting for the chain the store belonged to accounted for the association (*p* = 0.113), see Table 3.

**Table 3:** Association between store area SEI and promotion of most unhealthy foods

Socioeconomic index of the stores	Most unhealthy promotions n (%)	All other promotions n (%)	Total n (%)
More advantaged store areas	5294 (34.9)	9895 (65.1)	15,189 (50.7)
Less advantaged store areas	5907 (40.0)	8862 (60.0)	14,769 (49.3)
Total	11,201 (37.4)	18,757 (62.6)	29,958 (100.0)
Regression models for association between socioeconomic store index and promotion of most unhealthy foods		**OR (99% CI)**	**P**
Model 1 ^a^		1.25 (1.17, 1.33)^a^	< 0.001^a*^
Model 2 ^b^		1.00 (1.00 1.01)^b^	0.113^b^

^*^Significant association. Two-sided level of significance (*p* < .01)

^a^Value before adjusting for chain (A-G) the stores belong to as a confounding factor

^b^Value after adjusting for chain (A-G) the stores belong to as a confounding factor using multivariable logistic regression analysis

Our sensitivity analysis, removing the one chain with no stores in more disadvantaged areas, showed the results to be robust and remained significant.

## Discussion

### Proportion of promoted healthy and unhealthy food

The novelty of this study is that it evaluates the nutritional quality of food promotions in Sweden as well as comparing this by area-level SEI of the stores. We found that 66.8% of the promoted foods were from the unhealthy food categories. According to NNR, these items should be exchanged to a healthier alternative or limited in the diet. The most frequently promoted food category was ‘sugary beverages and food’.

Our finding that unhealthier food categories were promoted to a larger extent than the healthier categories is consistent with previous research. An international comparative study found that most countries promoted a high amount of discretionary compared to core food [15]. In a study from Australia, 43.3% consisted of discretionary foods, fats and oils and concluded that the promotions analyzed were not in line with the Australian dietary recommendations [18]. Another study conducted in Brazil followed the Pan-American Health Organization’s (PAHO) nutritional profile model and reported that all but 3.5% of the food promotions had a less healthy nutrient profile, where ultra-processed foods constituted 66.9% of the promotions [16]. Two studies from the Netherlands categorized approximately 70% of the promoted foods as unhealthy [26, 27], and four studies from United States found that most promoted foods were considered as unhealthy [17, 23–25]. Two studies reported that most promotions were for processed foods or simple carbohydrates, and few were high in dietary fiber or low in fat and sodium [17, 24]. It has also been observed that many promotions were for meat-based protein foods, where red meat and poultry constituted the majority [23, 25]. This is consistent with our study where processed meat and red meat constituted the second most promoted food category.

This study compares supermarket promotions with the NNR, a guiding framework for both dietary composition and macro- and micronutrient intake. The primary focus of the recommendations is to reduce the risk of diet-related chronic diseases by promoting a nutritious, low-energy diet and a physically active lifestyle to attain energy balance. Although the scientific evidence for total fat intake and health is limited, reducing total fat prevents excessive weight gain, which in turn is associated with health risks. Recommendations on fat intake aim both to reduce the proportion of total fat and to increase the quality of fat, where saturated and trans-fatty acids should be reduced and unsaturated fatty acids increase [21].

### Healthiness by store SEI

The unhealthiest group which included the food categories ‘processed meat’, ‘sugary beverages and food’, ‘salty food’ and ‘alcohol containing products’ was promoted to a larger extent in ad-sheets from supermarkets geographically located in more disadvantaged SEI areas. However, the association was fully explained by the supermarket chain to which the store belonged. This could indicate that certain chains that promote unhealthier food to a larger extent locate their individual stores in areas which are more socioeconomically disadvantaged.

One study conducted in Stockholm, Sweden investigated the difference of outdoor ultra-processed food advertisements in two diverse socioeconomic status (SES) areas [39]. They found a significantly higher proportion of ultra-processed food advertised in the less affluent SES area. A study from United States saw a significant difference in promotions between regions with high and low rates of obesity, where the regions with less obesity prevalence promoted more fruit while the regions with a higher obesity prevalence promoted more sweets [23]. This aligns with our study finding that less affluent areas are exposed to more unhealthy prompts. However, these other studies did not investigate the influence by chain as we have.

The social gradient in healthy dietary patterns is well established in high-income countries [40–44] and can to some extent be explained by factors in the surrounding food environment [1, 2, 45–47]. Promotion of unhealthy foods and soft drinks is contributing to the increase in childhood and adult overweight and obesity, thus the negative raise in non-communicable diseases (NCDs) globally [3, 9]. The results from this study exemplify important aspects of the Swedish consumer nutrition environment that do not support healthy and sustainable consumer choices. These factors might also contribute to the currently stagnant and widening health gaps [48]. To reduce social inequities in diet, healthy and acceptable food choices should be affordable for all consumers [49]. Price incentives in combination with other strategies, e.g. choice architecture and nudging, might be successful to make healthy options more attractive in the store setting [50].

An observation made during data input was that many of the ad-sheet promotions included in the analysis were not price-reduced. To what extent and why certain products end up in the ad of weekly offering without being price reduced was not investigated in this study but can be worth investigating in future research. To promote certain products in ad-sheets automatically increases their visual impact, which seems to be a successful nudging strategy to influence consumers’ food choices [2]. The observation that two-thirds of the promotions were classified in this study as unhealthy has been described as a ‘sludging’ as opposed to ‘nudging’ strategy [50].

### Public health implications

The Public Health Agency of Sweden has recognized the food environment as obesogenic and expressed that more knowledge is needed about the healthiness of the environment [51]. This study exemplifies weekly ads regularly promote less nutritious alternatives within several food categories. This may create further difficulties for consumers to make a healthy choice.

Many past health promotion interventions have focused on individual responsibility, although food choices result from an interaction between consumers’ own values and the surrounding food environment [1, 3, 6, 52]. It is therefore essential that the built food environment is compatible with dietary recommendations that support consumers in making healthy choices. Targeting environmental factors can be an effective upstream strategy because they often have far reaching effects that might contribute to narrowing health gaps [3, 10, 53–56].

### Strength and limitations

A strength with the study is that the food categories and four food healthiness groups align with NNR and WHO Europe’s nutrient profile model which are both reliable sources, established by a substantial amount of previous research. The use of these food categories also facilitates the process of making international comparisons of food marketing in future research projects [34].

A weakness is that ad-sheets from only one Swedish region were analyzed for the study and the results can therefore not be generalized to the whole of Sweden. However, the large region of western Sweden was covered, and two of the seven chains seem to be using the same ad-sheet version across all Swedish stores. Another limitation is the fact that data were collected from 2–29 March in 2020 so possible seasonal variation of the ads could not be taken into consideration.

## Conclusions

Our findings indicate that Swedish supermarkets promote a large proportion of unhealthy foods as classified by the Nordic Nutrition Recommendations. We also observe that certain national supermarket chains tend to locate their stores in more disadvantaged areas and promote a greater proportion of unhealthy foods in their weekly advertisements compared to the more advantaged areas. There is an urgent need for supermarkets to shift promotions toward healthier food items.

## Supplementary Information

**Additional file 1.**

## Data Availability

The datasets used during the current study are available from the corresponding author on reasonable request.

## Abbreviations

SEI: Socioeconomic index
NNR: Nordic Nutrition Recommendations
WHO Europe: WHO Regional Office for Europe
