# Correction to: Supermarket promotions in Western Sweden are incompatible with nordic dietary recommendations and differ by area-level socioeconomic index

**DOI:** 10.1186/s12889-023-16016-9

**Published:** 2023-06-20

**Authors:** Melissa Mjöberg, Lauren Lissner, Monica Hunsberger

**Affiliations:** School of Public Health and Community Medicine, Institute of Medicine, Sahlgrenska Academy, University of Gothenburg, Gothenburg, Sweden

**Correction to**: ***BMC Public Health*23, 795 (2023).**10.1186/s12889-023-15729-1.

Following publication of the original article, the authors identified an error in Fig. 1. The proportion of promoted ‘most unhealthy’ foods, the percentage of ‘beverages and foods with added sugar’ and ‘other foods’ were accidentally swapped. The correct figure is available in this correction article, the original article has been updated.

**Fig. 1:**
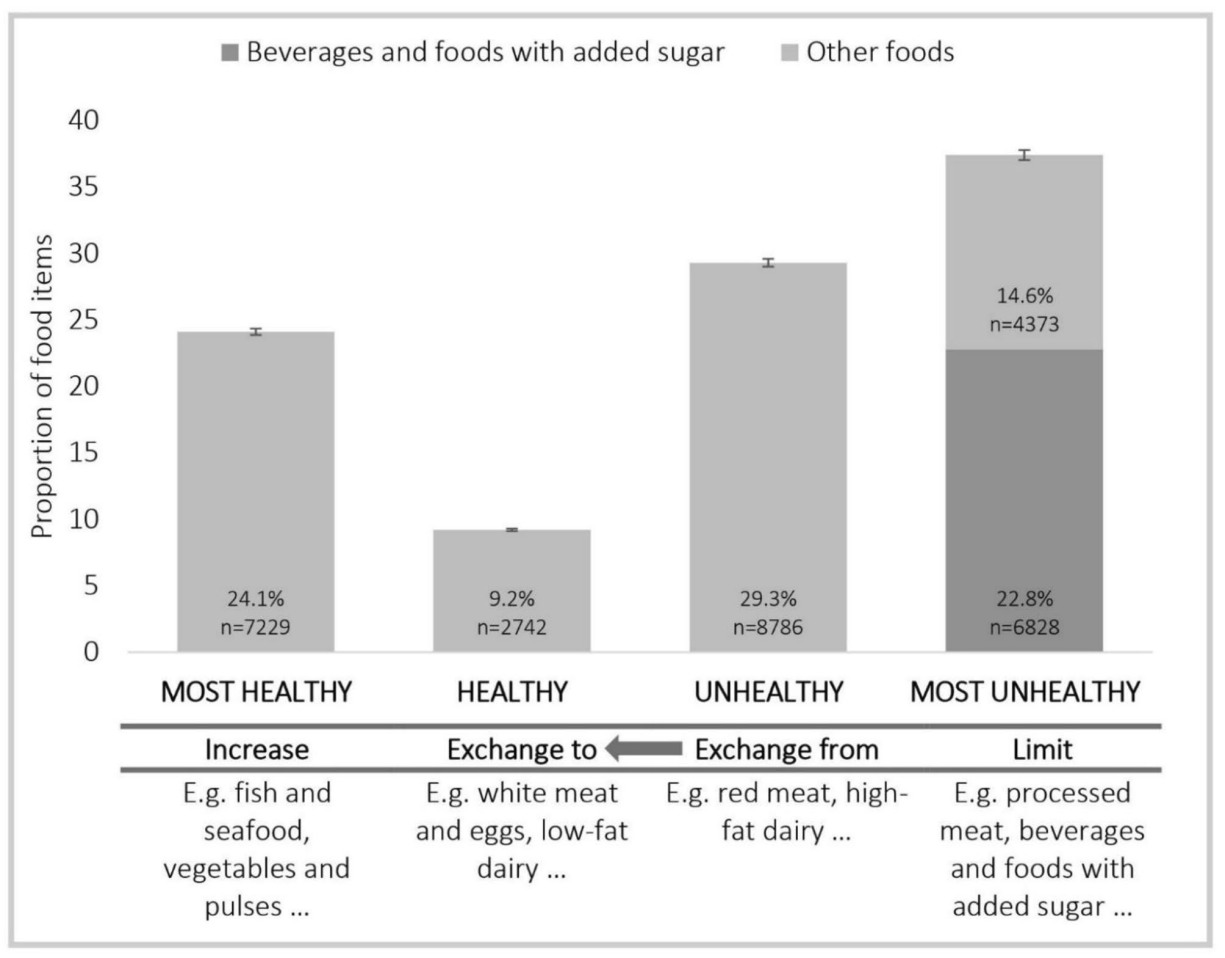
Proportion of promoted foods across the four health groups with corresponding recommendations

