# Supplementary material for: Supermarket promotions in Western Sweden are incompatible with Nordic dietary recommendations and differ by area-level socioeconomic index
Source: BMC Public Health. 2023 Apr 28;23:795. doi: 10.1186/s12889-023-15729-1 (PMC10148457; doi:10.1186/s12889-023-15729-1)
Supplement: Supplementary file 1 — Additional file 1. [file 12889_2023_15729_MOESM1_ESM.docx]

Appendix: Food categories inclusion conditions

| FOOD GROUPS | CONDITIONS FOR INCLUSION |
| --- | --- |
| 1. 1. MOST HEALTHY | |
| 1.1 Vegetables and pulses | - Including mushrooms and root crops. Fresh and frozen. Processed ok. |
| 1.2 Fruits and berries | - Fresh and frozen. Not processed but heated ok. |
| - 1. 1.3 Fish and seafood | - Fresh and frozen, including non-processed fish rye and caviar. - Processed ok if the product contains >50% fish or seafood. The product can contain sauce or similar and be breaded. Including processed caviar products. |
| - 1. 1.4 Nuts and seeds | - Nuts and seeds including peanuts. Not processed. |
| 1. 2. HEALTHY | |
| 2.1 Wholegrain cereals | - Hard bread. - Soft bread ≥*5% dietary fiber*. - Rice, couscous, bulgur *≥3% dietary fiber*. - Other grains, grain mixes *100% whole wheat and ≥6% dietary fiber.* - Breakfast cereals *≥55% whole wheat,* ≥6*% dietary fiber and ≤13% sugar whereas added sugar ≤9%.* - Pasta without filling *≥50% whole wheat and ≥6% dietary fiber*. |
| 2.2 Vegetable oils and vegetable oil-based fat spreads | - Vegetable oils and margarine with *≤20% saturated fat and ≤1% salt*. |
| 2.3 Low-fat dairy | - Milk without flavor *≤0,7% fat.* - Vegetable replacement products for milk and yoghurt *≤1,5% fat whereas ≤33% saturated fat.* If flavored yoghurt *≤4% sugar.* - Sour milk and yoghurt, natural *≤1,5% fat.* If flavored *≤1,5% fat and ≤4% sugar.* - Cream, crème fraiche, cooking yoghurt *≤5% fat.* If flavored *≤5% fat, ≤5% sugar and ≤0,8% salt.* Vegetable replacement products have the same requirements and that *≤33% saturated fat*. - Hard cheese *≤17% fat and ≤1,6% salt.* For vegetable replacement products *≤17% fat whereas ≤20% saturated fat and ≤1,5% salt.* Ok to be flavored*.* - Cottage cheese and other creamy cheese products *≤5% fat, ≤0,9% salt and ≤1% added sugar.* |
| 2.4 Low-fat sauces, dips and dressings | - Oil and vinegar based *≤20% saturated fat, ≤5% sugar and ≤0,8% salt.* - Dairy based *≤5% fat and ≤33% saturated fat, powder-sauce included*. |
| 2.5 White meat and eggs | - Fresh and frozen poultry. - Eggs. |
| 2.6 More nutritious ready-made and convenience products and composite dishes | - Products with ≥60% vegetable content and cannot include fish or meat. *Fat ≤10% whereas ≤33% saturated fat, ≤3% sugar and ≤1% salt.* The product can contain sauce or similar and be breaded. - Ready-made dishes which are made as a complete meal should be between 400-750 kcal and consist of ≥28%. - Root crops, legumes (peanuts excluded), other vegetables (potatoes excluded) or fruit and berries/100g product. Fat *≤33% with ≤10% saturated fat, ≤3% added sugar and ≤3,5% salt.* If it includes any of the cereals in 2.1 the requirements should be fulfilled as well. - Soups without meat and fish should include *≤50%* root crops, legumes (peanuts excluded), other vegetables (potatoes excluded) or fruit and berries/100g product. The requirements above for fat, sugar and salt should be fulfilled. - Composite dishes not made as a complete meal should include *≤50%* root crops, legumes (peanuts excluded), other vegetables (potatoes excluded) or fruit and berries/100g product. The requirements above for fat, sugar and salt should be fulfilled. |
| 1. 3. UNHEALTHY | |
| 3.1 Refined cereals | - Hard bread. - Soft bread *<5% dietary fiber.* - Rice, couscous, bulgur <*3% dietary fiber*. - Other grains, grain mixes <*100% whole wheat and <6% dietary fiber.* - Breakfast cereals *<55% whole wheat, <*6*% dietary fiber and sugars >13% whereas added sugars >9%.* - Pasta without filling *<50% whole wheat and <6% dietary fiber*. |
| 3.2 Butter and butter-based spreads | - Butter, similar products and products exceeding the requirements in 2.2. |
| 3.3 High-fat dairy | - Milk without flavor >*0,7% fat.* - Vegetable replacement products for milk and yoghurt >*1,5% fat whereas >33% saturated fat.* If flavored yoghurt >*4% sugar.* - Sour milk and yoghurt, natural *>1,5% fat.* If flavored >*1,5% fat and >4% sugar.* - Cream, crème fraiche, cooking yoghurt *>5% fat.* If flavored >*5% fat, >5% sugar and >0,8% salt.* Vegetable replacement products have the same requirements and that >*33% saturated fat.* - Hard cheese *>17% fat and >1,6% salt.* For vegetable replacement products >*17% fat whereas >20% saturated fat and >1,5% salt.* Ok to be flavored*.* - Cottage cheese and other creamy cheese products *>5% fat, >0,9% salt and >1% added sugar.* |
| 3.4 High-fat sauces, dips and dressings | - Oil and vinegar based *>20% saturated fat, >5% sugar and >0,8% salt.* - Dairy based *>5% fat and >33% saturated fat, powder-sauce included*. |
| 3.5 Red meat | - Fresh and frozen red meat. |
| 3.6 Less nutritious ready-made and convenience products and composite dishes | - All products exceeding the requirements in 2.6. |
| 1. 4. MOST UNHEALTHY | |
| 4.1 Processed meat | - Processed meat products. |
| 4.2 Beverages and foods with added sugar | - Fruit and vegetable juice. - Soda, energy drink, cider including light products and alcohol-free beer and wine; Chocolate milk, drink yoghurt and other sweet milk drinks. - Jam, marmalade and similar products. - Ice cream. - Chocolate and sugar confectionery, energy bars, and sweet toppings and desserts. - Cakes, sweet biscuits and pastries, other sweet bakery wares and dry mixes for making such. - Sugar, syrup, honey and sweeteners. |
| 4.3 Salt | - Snacks, processed nuts and seeds, potato chips, popcorn etc., fruit and nut mixes. |
| 4.4 Alcohol containing products | - Alcoholic beverages. |
